# Shift happens: trailing edge contraction associated with recent warming trends threatens a distinct genetic lineage in the marine macroalga *Fucus vesiculosus*

**DOI:** 10.1186/1741-7007-11-6

**Published:** 2013-01-23

**Authors:** Katy R Nicastro, Gerardo I Zardi, Sara Teixeira, João Neiva, Ester A Serrão, Gareth A Pearson

**Affiliations:** 1 CCMAR - CIMAR Laboratório Associado, Universidade do Algarve - Campus de Gambelas, Faro, 8005-139 Portugal

**Keywords:** Climate change, geographic boundaries, microsatellites, cryptic genetic erosion, sea surface temperature

## Abstract

**Background:**

Significant effects of recent global climate change have already been observed in a variety of ecosystems, with evidence for shifts in species ranges, but rarely have such consequences been related to the changes in the species genetic pool. The stretch of Atlantic coast between North Africa and North Iberia is ideal for studying the relationship between species distribution and climate change as it includes the distributional limits of a considerable number of both cold- and warm-water species.

We compared temporal changes in distribution of the canopy-forming alga *Fucus vesiculosus *with historical sea surface temperature (SST) patterns to draw links between range shifts and contemporary climate change. Moreover, we genetically characterized with microsatellite markers previously sampled extinct and extant populations in order to estimate resulting cryptic genetic erosion.

**Results:**

Over the past 30 years, a geographic contraction of the southern range edge of this species has occurred, with a northward latitudinal shift of approximately 1,250 km. Additionally, a more restricted distributional decline was recorded in the Bay of Biscay. Coastal SST warming data over the last three decades revealed a significant increase in temperature along most of the studied coastline, averaging 0.214°C/decade. Importantly, the analysis of existing and extinct population samples clearly distinguished two genetically different groups, a northern and a southern clade. Because of the range contraction, the southern group is currently represented by very few extant populations. This southern edge range shift is thus causing the loss of a distinct component of the species genetic background.

**Conclusions:**

We reveal a climate-correlated diversity loss below the species level, a process that could render the species more vulnerable to future environmental changes and affect its evolutionary potential. This is a remarkable case of genetic uniqueness of a vanishing cryptic genetic clade (southern clade).

## Background

Global climate change has a profound influence on distributional patterns of a wide variety of taxa causing species range shifts and population extinctions [1,2]. Poleward movements in latitude or increases in altitude have been observed in several geographic regions and for many species' ranges (for example, [3-6]) but the effects of contemporary climatic change at the so-called trailing edges have received considerably less attention.

Although several studies have addressed phylogeographic responses to millennial-scale climate variability [7], surprisingly few empirical studies have investigated how contemporary climate change on decadal scales impacts genetic diversity [2,8]. Climate-driven range reduction can have significant genetic and evolutionary consequences for surviving populations by decreasing genetic diversity and hindering a population's ability to adapt to future ecological disturbances (for example, [9]). Most studies investigating the effects of climate change on biodiversity consider a species as a unit and thus overlook intraspecific genetic variation. However, vulnerability to genetic depauperation depends on the geographical distribution of genetic diversity within a species; for those in which diversity is relatively homogeneous throughout the distributional range the genetic effect of range contraction is largely independent of which part of the range is lost (for example, [8,10]). However, genetic depletion will strongly depend on which part of the range is affected for species with a geographically skewed distribution of genetic diversity (for example, [11]).

Current geographical distribution of genetic diversity reflects both contemporary and historical events. Populations that are currently geographically peripheral may be particularly vulnerable to loss of genetic diversity because they are often restricted to decreasing habitat areas within unsuitable landscape. Their small size and prolonged isolation is accompanied by loss of within-population genetic diversity and increased inter-population genetic divergence due to increased random genetic drift, probability of suffering bottleneck effects, and reduced gene flow [12,13]. Previous climatic changes such as the glacial episodes of the Quaternary have left marked genetic signatures in present-day geographical distribution of genetic diversity. In particular, lower latitude regions where populations persisted through multiple glacial cycles (that is, glacial refugia), often host populations with higher genetic diversity than those in areas that were recolonized (for example, [14,15]). Importantly, marginal refugial populations that did not play a role in postglacial colonization processes have considerable conservation value because they may harbor unique genetic variation that is threatened by recent climate warming [16-18].

The stretch of Atlantic coast between North Africa and Iberia is ideal for the study of the relationship between marine species distribution and possible effects of climatic warming because a considerable number of both cold- and warm-water species reach their distributional limits within this stretch of coast (for example, [19-22]). In addition, several studies have described species distributional ranges and abundance clines since the middle of the 20th century in conjunction with abundant museum and herbarium data (for example, [22-30]), providing a solid historical background for studies aiming to describe changes in species distribution or abundance associated with environmental changes.

Surveys carried out in the last decade along the Atlantic Iberian Peninsula have correlated increasing sea surface temperature (SST) to striking expansion or contraction events [31-33]. SST is the prevailing factor controlling geographic distributions of many marine species at a latitudinal scale, either via direct effects on thermal tolerance or indirectly through changes in competition or predation dynamics [34-36]. While this is a recent worldwide trend, single-species response may be highly variable [5] and generalizations about poleward range shifts due to increasing temperature are not always possible [37,38].

In this study, the model species selected was *Fucus vesiculosus*, an intertidal canopy-forming brown alga. The availability of records on the species distribution along North African and Iberian Atlantic shores and of DNA from recently extinct populations provides an ideal opportunity to investigate the impact of climate-driven range reduction on the overall species genetic diversity.

We investigate shifts in the southern distribution endpoints of *F. vesiculosus *during the last three decades along this stretch of coast. Resulting biogeographic dynamics are compared with SST warming trends in order to correlate range shifts with the intensity of climatic change in the region. We further draw on genetic data from extant populations and others that have recently become extinct (previously sampled) in a phylogeographic approach to test the extent to which unique genetic variation may be lost as a result of climate change and range contraction.

## Results

### Past and present distributions and endpoints

**Figure 1:**
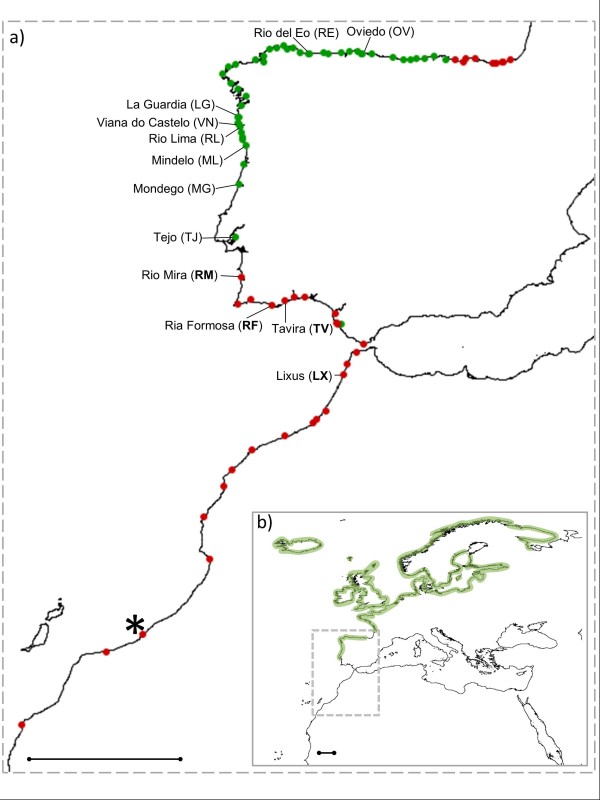
**Study locations**. (**a**) A map of the study area; dots are locations surveyed for *Fucus vesiculosus *during 2009-2011, presence or absence of species is marked in green and red, respectively; the asterisk (*) depicts the species historical southern distribution limit; names of population samples used for genetic analyses are reported, their codes are given in brackets and those in bold represent extinct populations; (**b**) a map depicting the complete northeastern Atlantic distribution of *F. vesiculosus*.

A literature review located the historical southern endpoint of *F. vesiculosus *in Southern Morocco at Khnifiss Lagoon (27°59'40.82"N-12°16'33.98"W; Figure 1). Its presence at this location was last reported in 1985-1986 and it was also confirmed by the Institut Scientifique, Département de Botanique et d'Ecologie Végétale in Rabat (personal communication by Dr. Ibn Tattou Mohamed; see Additional file 1).

**Figure 2:**
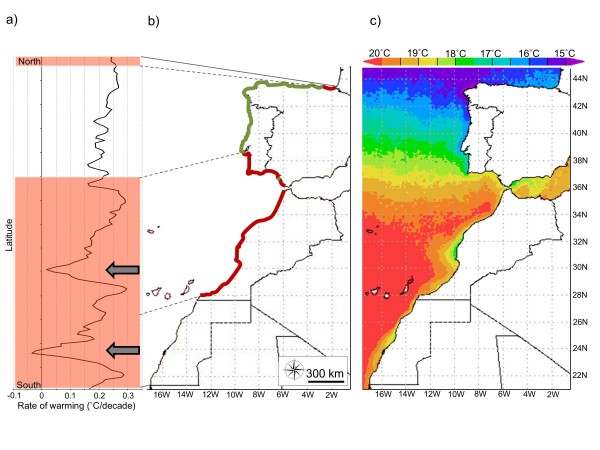
**Species distribution and temperature trends along the study area**. (**a**) Coastal SST warming data over the last three decades (°C/decade; 1982 to 2011) based on AVHRR, NOAA Optimum Interpolation ¼ Degree Daily Sea Surface Temperature Analysis data. Arrows indicate areas of intense upwelling. (**b**) Current and past distribution and endpoints of *F. vesiculosus *based on data from our field survey (2009 to 2011) and on literature information and herbarium collections, red and green lines are contractions and present distribution, respectively. (**c**) Monthly averaged (2009 to 2011) sea surface temperature; 4 km resolution based on MODIS-Aqua dataset. MODIS-Aqua, Moderate Resolution Imaging Spectroradiometer-Aqua; SST, sea surface temperature.

The southern endpoint is now positioned on the Portuguese west coast in the Tejo estuary (38°45'38.99"N, 8°56'28.43"W), representing an 11° latitude shift (1,250 km range contraction; Figure 2b). In addition, an isolated population was located in Cadiz-Puerto Real (36°31'27.53"N, 6°10'52.61"W) approximately in the middle of this range contraction and unattached vegetative fragments of adult *F. vesiculosus *were still observed entangled in *Spartina *grass in Ria Formosa (37°00'36"N, 7°59'31"W), although the previously permanent and attached populations have totally disappeared from this coastal lagoon.

Historically, *F. vesiculosus *populations were reported along the whole Northern Iberian coastline, with the latest presences at north eastern locations dated from 1951 to 1988. Currently, a limited retreat of 70 km within the Bay of Biscay has occurred and the present endpoint is at Colindres, eastern Cantabria (43°23'36.63"N, 3°27'50.15"W).

### Temperature trends

Temperature isotherms shifted polewards to higher latitudes (Figure 2c). This gradient is interrupted by areas of strong and persistent upwelling. In particular, in Morocco, the constant upwelling off Cape Ghir (centered at 31°N) generates a clear cold thermal intermission. Other upwelling cells are present to the south (Western Sahara), but fall outside the distributional range of *F. vesiculosus*. In contrast to North African shores, upwelling waters off north-west Iberian shores contribute to the SST gradient.

Over the last 30 years, a significant increase of coastal SST (°C/decade; Figure 2a) was observed throughout most of the entire Iberian and North African Atlantic coast. Coastal warming rates were not significant at relatively restricted areas within upwelling cells off Morocco (between 31°22' and 32°22') and Western Sahara (between 24°22' and 25°52'). In non-upwelling areas, coastal SST warming ranged from 0.092 to 0.297°C/decade, with an average of 0.214°C.

### Genetic analyses

Previously sampled *F. vesiculosus *collected in 2001 and 2002 from 12 locations, including four extinct populations (Figure 1), were genotyped at five microsatellite loci. The whole dataset (668 individuals genotyped) was highly polymorphic and the total numbers of alleles per locus were: 17 (locus 20; Additional file 2), 8 (locus 58; Additional file 3), 11 (locus 94; Additional file 4), 14 (locus 78; Additional file 5) and 9 (locus 38; Additional file 6).

Allelic richness (*Â*) varied between 6.28 and 3.08 with lowest values for ML, RF, TV and LX, while unbiased heterozygosity (*H*_E_) ranged between 0.381 and 0.587 with highest gene diversity values for RE, VN, RL and RM (location codes as reported in Figure 1). Observed heterozygosity (*H*_O_) was significantly different from *H*_E _in nine locations where heterozygote deficiency was detected, resulting in significant positive values of the inbreeding coefficient *F*_IS _(Table 1). The high values of *F*_IS _obtained were not locus dependent, indicating the markers used did not display technical issues such as null alleles. Unique alleles were detected for 8 of the 12 populations and varied between one and three.

**Table 1:** Genetic diversity of each population.

Location	N	*H*_E_	*H*_O_	*F*_IS_	*Â*_20 _± SD	UAN
OV	21	0.489	0.498	0.009	4.76±0.089	1
RE	29	0.587	0.401	0.336^b^	6.28±0.303	3
LG	48	0.420	0.342	0.197^b^	5.12±0.335	1
VN	94	0.583	0.509	0.132^b^	4.52±0.415	1
RL	48	0.571	0.377	0.349^b^	5.04±0.358	2
ML	48	0.486	0.388	0.213^b^	3.44±0.434	0
MG	96	0.457	0.437	0.050	4.20±0.200	2
TJ	96	0.381	0.346	0.096^a^	4.16±0.297	1
**RM**	48	0.580	0.318	0.460^b^	4.48±0.303	0
**RF**	48	0.410	0.380	0.083	3.44±0.219	0
**TV**	48	0.433	0.361	0.177^b^	3.44±0.434	1
**LX**	44	0.323	0.218	0.335^b^	3.08±0.228	0

^a^ <0.05 and ^b^ <0.001 using 10,000 permutations. Codes correspond to locations in Figure 1 and are ordered from north to south, bold letters are extinct populations; N, sample sizes; *H*_O _and *H*_E_, observed and expected heterozygosity; *F*_IS_, inbreeding coefficient with significant values. *Â*_20_, mean allelic richness normalized to the smallest sample size; SD, standard deviation of *Â*_20_; UAN, unique allelic number.

Strong differentiation among populations was found as estimated by pairwise genetic differentiations *F*_ST _and *D*_EST _values (10,000 permutations, *P *< 0.01 for all comparisons, see Additional file 7; all confidence intervals do not overlap with zero, see Additional file 8). Pairwise differentiation was lower within two subgroups (hereafter 'northern' group: OV, RE, LG, VN, RL, ML; and 'southern' group: MG, TJ, RM, RF, TV, LX; location codes as reported in Figure 1). However, *D*_EST _estimates were particularly high between RE and all the other locations whereas LX and TJ had the lowest divergence.

**Figure 3:**
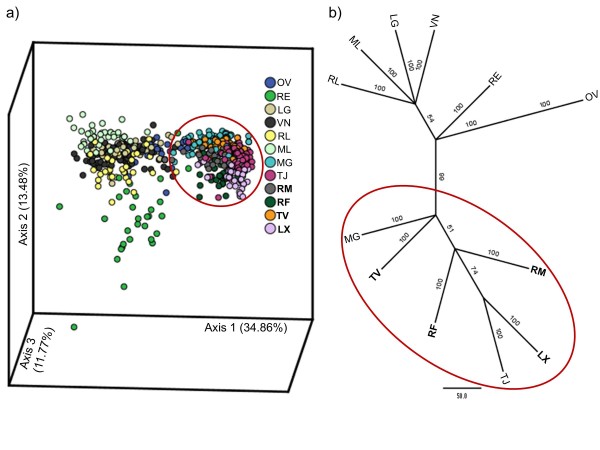
**Correspondence Analyses and Neighbor-Joining tree**. (**a**) Correspondence Analyses based on allele frequencies at five microsatellite loci. Percentages of inertia are shown between parentheses for each axis. (**b**) Neighbor-Joining tree inferred from Cavalli-Sforza and Edwards's pairwise distances; only bootstrap values higher than 50 are shown. Codes correspond to locations in Figure 1, locations belonging to the southern lineage are encircled and extinct populations are shown in bold.

**Figure 4:**
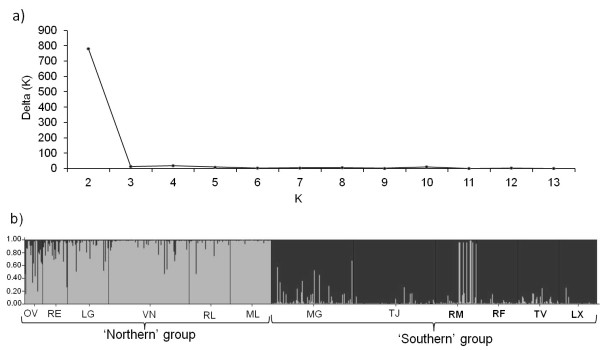
**Histogram of STRUCTURE assignment of genotypes to clusters**. (**a**) Plot of deltaK (an ad hoc statistic based on the rate of change in the log probability of data between successive *K *values and used to predict the real number of clusters [101]) where the modal value of the distribution is considered as the highest level of structuring, in our case two clusters. (**b**) Genetic structure of populations defined by STRUCTURE software where each vertical bar represents an individual. The membership for each individual is indicated by partitioning the corresponding line into several parts with different colors. Codes correspond to locations in Figure 1 and extinct populations are shown in bold.

On the basis of the dimensions scores of the Correspondence Analyses (CA), populations were grouped into two major clusters corresponding to the northern and the southern subgroups (Figure 3a). The first axis explains mostly the variance between these two clades (34.86%), while the second and third axes mainly illustrate the differentiation within the two subgroups (13.48% and 11.77%, respectively). Overall, the northern group had a higher inter-population diversity, which is illustrated by the more scattered distribution of the points in the plot. The CA is consistent with the tree topology based on allele frequencies using Cavalli Sforza and Edwards' chord distance (Figure 3b), where two main branches were resolved. The first of these two subgroups comprised six populations (northern group). A second subgroup (southern group) consisted of six populations, two of which are still present at the locations while the rest are now extinct. This major genetic subdivision was supported by the results obtained with STRUCTURE, which revealed the most significant increase at ΔK = 2 clusters (Figure 4a), thereafter ΔK remained unchanged. We concluded that K = 2 is the most likely number of genetic clusters (Figure 4b).

The northern group had many unique alleles, but the southern cluster including the now extinct populations also displayed unique alleles. This cluster suffered diversity loss with the recent extinctions, but still retains unique diversity (three unique alleles detected in five loci assessed, at MG, TJ; Table 2).

**Table 2:** Genetic diversity of each cluster.

	*H*_E_	*Â *_280/190 _± SD *Â *_280/190_	UAN
Northern cluster	0.6281	11.9/11.2±0.11/0.26	16
Southern cluster-past	0.545	9.08/n.a±0.3/n.a	4
Southern cluster-present	0.4934	n.a./7.8 ± n.a./0	3

Northern cluster include locations OV, RE, LG, VN, RL, Ml, southern cluster-past include MG, TJ, RM, RF, TV, LX while southern cluster-present is formed only by extant populations of this latter clade (MG and TJ). *H*_E_, expected heterozygosity; *Â*_280/190_, mean allelic richness normalized to the smallest sample size of the pooled clusters (N = 280/190); SD *Â*_280/190_, standard deviation of *Â*_280/190_; UAN, unique allelic number.

## Discussion

A rapid and extensive range contraction of the southern border of a marine intertidal species occurred, with a northward shift of the historical limit of about 11° latitude. This remarkable distributional shift consists of a latitudinal reduction of about 23% of the entire species range along eastern Atlantic shores and has resulted in the erosion of a cryptic evolutionary lineage at this trailing edge. Additionally, the range contraction is consistent with significant increases in SST throughout most of the coastal area studied.

### Range shift in southern Iberia

Systematic shifts in geographical ranges of species and increased extinction risk has emerged as one of the most pervasive biological responses to recent anthropogenic climate warming. The general trend shows range expansion towards higher latitudes; however, redistribution dynamics at southern range margins are less clear (for example, [5]). While on land, southern or 'trailing' range boundaries of terrestrial ectotherms may not shift consistently towards the poles with climate warming, marine species ranges match more closely their limits of thermal tolerance. Thus, range shifts will show a more predictable southern range contraction in the marine realm [4].

Extinction of southern edge populations along the Iberian Peninsula has been reported for other marine cold temperate species [17]. Another study conducted along this stretch of coast highlights the complexity in making generalizations about range shifts for intertidal species [37]. The extensive historical records reviewed in our study indicate that until the late 1980s abundant populations of *F. vesiculosus *were still present along the entire Moroccan and Iberian Atlantic coasts, while in the early 2000s extensive populations of this alga still persisted in southern Portugal. When these records are compared with our more recent surveys (2009 to 2011), a major contraction of the southern range margin of this keystone intertidal species (that is, from southern Morocco to central Portugal, latitudinal reduction of more than 1,200 km) is revealed. Taken together with past studies, there is a strong indication that the northward distributional shift along the Portuguese coast occurred very recently, probably within the last decade. This range contraction is an order of magnitude larger than the range shifts recorded for other species over the last 50 years along the Northeast Atlantic (for example, [31,37]).

Trends in SST indicated that the large scale disappearance of southern populations of *F. vesiculosus *is correlated with warming of North African and southwest Iberian coastal waters, with steady year-round increases averaging >0.2°C/decade over the last three decades. Seasonal thermal extremes might also play a crucial role in defining the distribution of species through sudden population declines or die-offs [39]. However, the key pressure exerted by increasing average SST may result from sub-lethal rather than catastrophic effects. In the North-Eastern Atlantic, the distributional limits of most warm and cold-water algal species seem to be set by sub-lethal effects of water temperature, through reduced reproduction and growth, rather than by lethal effects [34,40]. Several other factors indirectly related or unrelated to changes in temperature may be responsible for the observed distributional shifts. These include biotic interactions (for example, grazing and competition [41]) and near-shore abiotic or anthropogenic effects (for example, pollution, coastal erosion, wave [38,42]). Moreover, seaweed survival, growth, and reproduction are known to be largely affected by latitudinal variations in nutrient supply [43,44]. However, mean or maximum water temperatures appear to be the best overall predictors of mortality events in the intertidal zone and the main determinants of large scale range contractions (for example, [36,45-47]) as opposed to maximum air temperatures. Intertidal organisms are essentially at the same temperature as water during high tide, while thermal conditions experienced during low tide are more likely to be affected by individual physiological [48,49] and behavioral [50] capacities and by the heterogeneity of intertidal microhabitats [51,52].

The abundance of species is generally expected to peak at the center of their distributional range and decline with adverse conditions towards the range limits (for example, [53]). However, several empirical studies have challenged the 'abundant center' view of species distribution [54], such as in the intertidal zone where localized variations in the thermal environment due to climatic and tidal interactions disrupt monotonic latitudinal temperature clines [54-56]. In particular, delivery of cold waters by upwelling is not directly correlated with atmospheric processes, providing an opportunity for regional de-coupling of global warming and the creation of refugial areas where intertidal communities can escape the effects of projected climatic changes [57,58]. Strong upwelling events off the northwest Iberian coastline reinforce latitudinal temperature gradients [32], while local upwelling further south (for example, the Cape Ghir region in Morocco, 31°N) disrupts latitudinal thermal clines. The latter constitutes one of the most persistent (year-round) upwelling cells along the northwest African coast [59]. The SST data clearly indicate that the upwelling region around Cape Ghir has not experienced significant warming, in contrast to adjacent non-upwelling areas (but see below). As a result, and in contrast to the Portuguese coastline, this upwelling cell disrupts the regional thermal latitudinal cline. Despite suggestions that upwelling could provide refugia from the thermal stress of ocean warming [57,58], our results show that *F. vesiculosus *populations affected by cold upwelled waters have also undergone extinction. In Cape Ghir, warming rates show variation at monthly timescales, with a significant SST increase of about 0.28°C/decade in March (http://coastalwarming.com[60]) and maximum SST peaking occasionally over 24°C [61]. Although monthly SST anomalies and high temperature events are less frequent and intense than in contiguous non-upwelling areas, they might be strong enough to cause extinction. Furthermore, effects of climate variability can be aggravated if isolation limits gene flow between populations, increasing genetic loss to drift with consequent loss of evolvability to cope with future environmental change [9]. While northern populations are well connected by continuous patches of *F. vesiculosus*, populations at the southern edge were fragmented and spatially isolated prior to extinction, potentially limiting population size and increasing susceptibility to drastic demographic and genetic variation.

Interestingly, patches of *F. vesiculosus *persist within the Bay of Cadiz, along the retreating front. This represents a restricted (extending less than 1 km along the coast) population confined to very sheltered areas within the bay (personal observation). The forecasted warming trend suggests that this small and isolated relict population faces a high risk of extinction, and this prediction is further supported by the extinction of nearby (less than 10 km distant) populations (for example, La Caleta and Playa de la Victoria).

### Range shifts in northern Iberia

Along eastern Atlantic shores, *F. vesiculosus *extends its northern distributional limit in Norway, also inhabiting the northernmost and brackish regions of the Baltic Sea and in the White Sea into brackish tidal marshlands. Within the range of *F. vesiculosus*, there is a well-documented gap along the SW French coast (French Basque Country and Gascony) due to lack of rocky substratum [62]. In contrast to the southern clade, our results indicate that the present distributional range of the northern lineage in Iberia has been affected minimally, with a contraction of approximately 70 km at the southern limit of this gap (Spanish Basque Country). Notably, *F. vesiculosus *individuals from other regions in Europe are genetically closer to the northern Iberian clade identified in our study by the same markers [63]. A graphical model based on IPCC (Intergovernmental Panel on Climate Change; [64]) scenarios predicted the range decline observed in our study [65]. This model extends its forecast until 2050 predicting that, by 2025, cold water species, including *F. vesiculosus*, will disappear completely from the Bay of Biscay.

In Northern Spain, recent distributional declines have also been reported for *Fucus serratus *[66], *Himanthalia elongata *[67] and three kelp species [68], most probably caused by significant increases in SST [69] and unusually intense warm inflow of seawater during autumn-winter in 2006 [70]. Interestingly, despite these warming trends, present SST at the eastern limit of *F. vesiculosus *in Northern Spain is lower than that at the southern limit in Portugal. This suggests that the two genetic lineages have different thermal tolerances or that distinct factors are setting their distribution limit. Indeed, different genetic lineages or populations inhabiting different areas within a species' distributional range can show diverse resistance or resilience to abiotic stresses (for example, [49,71]). For example, along Atlantic European shores, southern edge populations of *F. serratus *are less resilient to desiccation and heat shock than central populations [49]. Future experimental approaches could allow explicit tests for signs of local adaptation between southern and northern lineages of *F. vesiculosus*.

### Genetic diversity under threat

Reduction in genetic variation gives independent evidence of the severity of population and habitat contraction. Estimating losses in genetic diversity associated with habitat contraction and environmental change is one of the key challenges in biodiversity research. The intensity of genetic loss largely depends on the geographical distribution of genetic diversity within the species, including cryptic diversification. Our analyses revealed two clusters, coinciding with more northern and southern locations, distinguished by several analyses based on allele frequencies, and containing unique alleles within each group, in the five microsatellite loci here used. The differentiation between northern and southern lineages in *F. vesiculosus *was clearly established using a large number of distinct genetic markers including 13 protein coding genes [72] and 35 SNP markers [73]. The separation between these lineages of *F. vesiculosus *is older than the divergence between other more recently evolved species within the genus *Fucus *[71], highlighting their conservation value. The same multigene assays [70,71] could not be performed on the old DNA left from the extinct populations here studied, thus our genetic analyses of the extinct populations are only based on five microsatellites, yet these were sufficient to reveal unique alleles.

The divergence between northern and southern lineages does not appear to be absolute, since a few individuals appear to occur in mixed populations (STRUCTURE analysis; for example, RE, OV, RM). Additionally, individuals from RE, not only diverge from southern populations, but emerge as the most distinct within the northern clade (CA analysis) with no apparent geographical explanation. This appears geographically unexpected, but patterns of genetic structure and differentiation are not necessarily maintained by prominent ecological/oceanographic barriers to dispersal [74]. For example, in *Fucus ceranoides*, distant (but relatively similar) populations do not necessarily exchange more migrants than closer (but relatively distinct) populations [18]; historical patterns of extinction and colonization may play a more important role than ongoing gene flow in determining the extent of genetic divergence between extant populations.

Presently, the distributional range of the southern clade is restricted within approximately two degrees of latitude (17% of the historical range) and southern intra-specific genetic diversity is represented by few extant populations under threat from climatic warming. Given that predictions of future climate indicate a further rise in SST [75], there is a real risk of extinction of the southern phylogeographic distinctiveness owing to climatic shifts. In contrast, for long-term conservation purposes, it is reasonable to assume that, despite the relatively small range decline, the diversity of the northern clade is not under immediate risk of reduction because populations of this same genetic group are still present throughout Northern Europe.

Past and present distributional ranges of the southern lineage are characterized by markedly higher SST than those of the northern lineage; it can be hypothesized that the existence of this lineage is explained by local adaptation or phenotypic plasticity to withstand warmer waters. Our multi-locus genetic data represent neutral genetic change and do not necessarily reflect evolutionary responses to selection. However, divergent lineages often arise in areas of speciation (for example, [76,77]). Indeed, recent data indicate that the hermaphroditic species *F. virsoides, F. spiralis *and the recently-described *F. guiryi *[78] are derived from a dioecious ancestor sister to the southern clade of *F. vesiculosus *[72]. The potential loss of the cryptic southern *F. vesiculosus *lineage could end ongoing diversification (or speciation) processes and compromise the adaptive potential of the species as a whole in the face of future global warming.

In this study, genetic diversity of *F. vesiculosus*, as expressed by both gene diversity and allelic richness, was higher in northern Iberian populations than in the southern lineage. Higher genetic diversity in the northern Iberian region can be a signature of higher temporal stability of large populations while the opposite, in the southern region, could be the result of lower population sizes and/or temporal variability (that is, bottlenecks, extinctions/recolonizations) of the trailing edge populations. Higher diversity could also result from secondary contact between distinct lineages and/or from hybridization and introgression. While *F. vesiculosus *co-occurs with other fucoids throughout the majority of its range, including Northern Iberia, Southern Iberian individuals occur in allopatry. Therefore, hybridization events are likely only within and to the north of the contact zone between the sympatric and allopatric ranges [63]. Given the apparently small population sizes (small patches) of the populations of the southern group, which are, thus, prone to drift and bottlenecks, and their geographical isolation (separated by tens to hundreds of km), it is perhaps surprising that genetic diversity, although lower, still remains so close to northern levels. Regardless of the cause behind the current within-clade diversity, their genetic distinctiveness implies that the total diversity of *F. vesiculosus *would be significantly reduced should these last remaining populations also become extinct.

## Conclusions

A remarkable climate-induced species range reduction is driving a cryptic genetic clade (southern clade) to extinction. This clade occurs under stressful and selective environmental conditions and is, therefore, a potential pool of adaptive traits for the species; losing it could thus reduce the adaptive potential of the species, a serious conservation concern. However, the recent loss of most of the populations in this lineage suggests a more drastic scenario where their genetic distinctiveness has not been able to rescue them from extinction. Although low diversity within small marginal populations can reduce local adaptive capacity [47], here, even the southern clade hotspot of diversity (Rio Mira - RM) went extinct.

In addition to genetic erosion, the elimination of *F. vesiculosus *populations is likely to have other immediate ecological consequences. This is a dominant intertidal ecosystem engineer that influences coastal species richness by modifying habitats, increasing spatial complexity and facilitating the presence of other species [79,80]; thus, its large-scale disappearance may modify and decrease ecosystem complexity, reducing diversity and abundance of associated species (for example, Baltic Sea [81]), with potential effects on all trophic levels.

## Methods

### Past and present distributions and endpoints

Data on the past distribution and southern endpoint of *F. vesiculosus *along Atlantic North African and Iberian coastlines were gathered from a comprehensive literature review and information from herbarium collections. Data for the last decade also included personal records and associated sample collections made by our team in multiple surveys of the Iberian and Moroccan coastline for genetic sampling of this species for previous studies [63,72,82,83] and of species that co-occur in the same habitats [17,84,85]. Published literature was screened up until May 2012 in searches using Google Scholar and ISI Web of Knowledge and based on combinations of the following keywords: *Fucus, vesiculosus*, Morocco, Iberia, Iberian Peninsula, Portugal, Spain, France, Atlantic, Western Sahara, distribution, herbarium, Fucales, Fucophyceae. In addition, literature reported in AlgaeBase (http://www.algaebase.org) was screened and searched up until 16 May 2012. Sampling artifacts may bias range shift studies [86] because (a) the spatial and temporal scale of surveys are limited, and (b) taxonomical information and ecology of species are poorly known. We expect these limitations to have minimal effects on our data because (a) the entire stretch of coast examined in the present study has been the object of extensive research providing a fundamental dataset of the spatial and temporal extent of the species' geographic range; and (b) *F. vesiculosu*s is a scientifically popular species with well-known biology. It has the clear distinctive trait of possessing air bladders on the thallus, diagnostic for this species, and presents separate sexes, in contrast with the only other congeneric species occurring southwards from North Portugal. In addition, museum and literature records did not show any discrepancy. Therefore, we are confident about the quality of the points of reference used in this study.

Current distribution and endpoints were obtained based on an extensive field survey thoroughly covering the entire past distribution of *F. vesiculosus*. The survey was carried out during low spring tides between 2009 and 2011 on rocky intertidal shores along the Atlantic North African and Iberian coastlines. Eighty-four moderately wave exposed and estuarine locations were sampled from Hondarribia (Spain, 43°21'N 1°47'W) to Dakhla (Western Sahara, 23°41'N 15°55'W), covering a latitudinal extension of more than 2,150 km. Locations of the survey were chosen based on previous reports of the presence of *F. vesiculosus *(herbarium, DNA samples and literature data). Five additional locations were only surveyed in 2009 to 2011, of which two were in Western Sahara (Boujdour and Dakhla), south of the historical southern limit.

Most of the locations were visited at least twice, covering winter and summer months, and each location included two sites from 500 m to 1 km apart. At each site, two observers performed searches lasting approximately 60 minutes across all microhabitats present.

### Temperature trends

Monthly averaged SST data between January 2009 and December 2011 with a 4 km resolution were retrieved from the Moderate Resolution Imaging Spectroradiometer-Aqua (MODIS-Aqua) dataset available from the National Aeronautics and Space Administration (NASA) Goddard Earth Sciences (GES) Data and Information Services Center (DISC). Visualization was performed using Giovanni, a web-based application developed by the GES DISC [61].

Coastal SST warming data over the last three decades (°C/decade from January 1982 to December 2011) were obtained from the Worldwide Coastal Warming Assessment project website at http://www.coastalwarming.com/index.html[60]. This project uses Advanced Very High Resolution Radiometer (AVHRR), NOAA Optimum Interpolation ¼ Degree Daily Sea Surface Temperature Analysis data (Reynolds OI V2 SST data) acquired from the NOAA-National Climatic Data Center (http://www.ncdc.noaa.gov).

Note that, although warming rates at coastal and ocean locations often depend on the selected spatial and temporal scales, our SST trends are comparable with those reported in other studies that focused on different periods and spatial resolutions [31,59,87,88].

### Genetic data

Previously sampled *F. vesiculosus *collected in 2001 and 2002 from 12 locations, including four extinct populations, were used for genetic analyses (Figure 1). DNA extraction used the DNeasy™ Plant Mini Kit (Qiagen, Hilden, Germany) and five polymorphic microsatellite loci, L20, L58, L38, L94, L78 [89] were amplified and genotyped following the methods described in Perrin *et al. *[83].

### Data analyses

Observed (*H*_O_) and expected (*H*_E_) heterozygosities were estimated, and deviations from Hardy-Weinberg equilibrium were tested for significance with 10,000 permutations, using GENETIX 4.05 software (Belkhir *et al. *1996-2004), applying q-value correction for multiple tests [90]. Allelic richness (*Â*) was estimated for each population separately, standardized to 20 individuals to account for differences in sample size, using the StandArich package and R 2.10.1 software. Plot of allelic frequencies for each location and marker was performed with the above package.

To depict the global genetic variation among the samples, a CA based on the matrix of individual genotypes identified with five nuclear markers was performed using the factorial correspondence analysis (FCA) procedure on populations implemented in GENETIX 4.05 [91] and graphically visualized with IBM^® ^SPSS^® ^20 software.

*F_ST _*and confidence intervals were estimated between pairs of populations with the estimator *θ *[92], and computed using the R package 'DiveRsity' [93]. Additionally, pairwise population differentiation was calculated as estimates of Jost's D_EST _[94] using SPADE software [95]. Significance was tested using 10,000 random permutations of the individuals between samples with a threshold adjusted using q-value correction for multiple comparisons.

The Cavalli-Sforza and Edwards' chord distance [96] was computed (with GENDIST), as this measure has been shown to generate higher probabilities of obtaining the correct tree topology [97]. Neighbor-joining was used to assemble the tree in NEIGHBOR with bootstrap re-sampling (10,000 replications) executed using SEQBOOT and CONSENSE. All programs are part of the software package PHYLIP 3.69 [98]. The output was visualized in FIGTREE 1.3.1. [99] and edited in Adobe^® ^Illustrator^® ^CS4 14.0.0 (Adobe System Inc.).

STRUCTURE 2.3.3. software [100] was used to estimate the most probable number of population clusters (K). The analysis was run without prior information on populations, assuming correlated allele frequencies and admixture. The number of possible Ks assessed was 1 to 13 (maximum number of populations plus one) and 20 independent runs with 100,000 Markov Chain Monte Carlo (MCMC) iterations and 10,000 burn-in were performed at each K to calculate ΔK as in Evanno *et al. *[101]. CLUMPP software [102] was subsequently used to find the optimal alignment of the 20 replicate cluster analyses of the same K. The mean membership matrix across replicates was plotted with the program DISTRUCT [103].

Finally, to compare the level of diversity within clusters, estimates of *Â, H_O _*and *H_E _*were calculated for the different clusters determined by STRUCTURE analyses by pooling all populations from each cluster.

## Abbreviations

*Â*: allelic richness; CA: correspondence analyses; F_IS_: inbreeding coefficient; F_ST_: genetic differentiation; FCA: factorial correspondence analysis; H_E_: expected heterozygosity; H_O_: observed heterozygosity; K: population clusters; MODIS-Aqua: Moderate Resolution Imaging Spectroradiometer-Aqua; SNP: single nucleotide polymorphism; SST: sea surface temperature.

## Competing interests

The authors declare that they have no competing interests.

## Authors' contributions

GIZ, KRN, EAS and GAP conceived and designed the study. EAS and GAP provided distribution records and genetic data for extinct populations. GIZ and KRN gathered and analyzed temperature and distribution data. ST and KRN analyzed genetic data and all authors interpreted the data. KRN and GIZ wrote the article, which was critically revised by EAS and GAP. All authors read and approved the final version.

## Supplementary Material

Additional file 1 **List of locations surveyed**. Name and coordinates of locations surveyed during 2009-2011, ordered from north to south. Presence/absence of the species and latest known year of its presence is also reported. Click here for file

Additional file 2 **Allele frequencies for locus L20**. Codes correspond to locations in Figure 1, locations belonging to the southern lineage are encircled and extinct populations are shown in bold. The actual values of frequencies are represented by dots of varying diameter: allele codes are indicated on the x axis and population names on the y axis. Click here for file

Additional file 3 **Allele frequencies for locus L58**. Codes correspond to locations in Figure 1, locations belonging to the southern lineage are encircled and extinct populations are shown in bold. The actual values of frequencies are represented by dots of varying diameter: allele codes are indicated on the x axis and population names on the y axis. Click here for file

Additional file 4 **Allele frequencies for locus L94**. Codes correspond to locations in Figure 1, locations belonging to the southern lineage are encircled and extinct populations are shown in bold. The actual values of frequencies are represented by dots of varying diameter: allele codes are indicated on the x axis and population names on the y axis. Click here for file

Additional file 5 **Allele frequencies for locus L78**. Codes correspond to locations in Figure 1, locations belonging to the southern lineage are encircled and extinct populations are shown in bold. The actual values of frequencies are represented by dots of varying diameter: allele codes are indicated on the x axis and population names on the y axis. Click here for file

Additional file 6 **Allele frequencies for locus L38**. Codes correspond to locations in Figure 1, locations belonging to the southern lineage are encircled and extinct populations are shown in bold. The actual values of frequencies are represented by dots of varying diameter: allele codes are indicated on the x axis and population names on the y axis. Click here for file

Additional file 7 **Genetic differentiation between pairs of populations**. Codes correspond to locations in Figure 1 and are ordered from north to south, bold characters are extinct populations. Genetic differentiations (*F_ST_*) were estimated with the estimator *θ*, and are reported above the diagonal while Jost's *D*_EST _are reported below the diagonal. All values are significant at *P *<0.001 after multiple test correction. Click here for file

Additional file 8 **Confidence intervals of the genetic differentiation between pairs of populations**. Codes correspond to locations in Figure 1 and are ordered from north to south, bold characters are extinct populations. Confidence intervals of genetic differentiation (*F_ST_*) are reported above the diagonal. Click here for file
