# Supplementary material for: Shift happens: trailing edge contraction associated with recent warming trends threatens a distinct genetic lineage in the marine macroalga Fucus vesiculosus
Source: BMC Biol. 2013 Jan 23;11:6. doi: 10.1186/1741-7007-11-6 (PMC3598678; doi:10.1186/1741-7007-11-6)
Supplement: Additional file 1 — List of locations surveyed. Name and coordinates of locations surveyed during 2009-2011, ordered from north to south. Presence/absence of the species and latest known year of its presence is also reported. [file 1741-7007-11-6-S1.DOCX]

**Table A1 - List of locations surveyed**

Name and coordinates of locations surveyed during 2009-2011, ordered from north to south. Presence/absence of the species and latest known year of its presence is also reported. Historical and current endpoints are in bold while *depicts the small and restricted population outside present southern endpoint. In brackets, reference to the bibliographic data (and references therein) used to assert the year: **(a)** Margalet, J.L., Almaraz, T., Navarro, M.J., Pérez-Ruzafa, I.M. (1993). Mapas de distribución de algas marinas de la Península Ibérica. III. *Fucus ceranoides* L., *F. serratus* L., *F. spiralis* L. y *F. vesiculosus* L. (Fucales, Fucophyceae). Botanica Complutensis 18: 267-290; **(b)** Fishet-Piette, E., Duperjer, R. (1963). Situatione basque en 1961 et 1962. Bulí. Centudes Rech. Sci. Biarritz 4:407-416; **(c)** Moalic, Y., Arnaud-Haond, S., Perrin, C., Pearson, G.A., Serrão, E.A. (2011). Travelling in time with networks: Revealing present day hybridization versus ancestral polymorphism between two species of brown algae, *Fucus vesiculosus* and *F. spiralis*. BMC Evolutionary Biology 11: 33; **(d)** Lima, F. P., Ribeiro, P. A., Queiroz, N., Hawkins, S. J., Santos, A. M (2007). Do distributional shifts of northern and southern species of algae match the warming pattern? Global Change Biology 13:2592-2604 – according to personal observations of Pearson, G. A. & Serrão, E. A and Berecibar, E., this record of *F. vesiculosus* is likely to be a misidentification of *F. spiralis*, the Fucus species that at that time occurred at that site. Moreover, Lima et al. [[37](#_ENREF_37)] has focused on the Portuguese open coast, excluding from the study the habitat of southern *F. vesiculosus*, estuaries and coastal lagoons [[26](#_ENREF_26), [104](#_ENREF_104), [105](#_ENREF_105)] and southern Spanish and north African populations. Their sampling was performed in the early 2000s when the species *F. vesicul*osus was still present along the Algarve (southern Portugal) coast in the Arade estuary, the Ria Formosa coastal lagoon and the Guadiana estuary, and further south in Morocco (our records and DNA sample collections, Table SM1); **(e)** Machado, S. (2007) The origin of *Fucus vesiculosus* in the Ria Formosa. Honours thesis in Biochemistry. University of Algarve, Portugal.; **(f)** Pearson, G.A., Hoarau, G., Lago-Leston, A., Coyer, J.A., Kube, M., Henckel, K., Serrão, E.A., Corre, E., Stam, W.T., Olsen, J.L. (2010). An expressed sequence tag (EST) analysis of the intertidal brown seaweeds *Fucus serratus* (L.) and *F. vesiculosus* (L.) (Heterokontophyta, Phaeophyceae) in response to abiotic stressors. Marine Biotechnology, 12:195-213; **(g)** Perrin, C., Daguin, C., Van de Vliet, M., Engel, C.R., Pearson, G.A., Serrão, E.A. (2007). Implications of mating system for genetic diversity of sister algal species: *Fucus spiralis* and *Fucus vesiculosus* (Heterokontophyta, Phaeophyceae). European Journal of Phycology. 42: 219-230; **(h)** Coyer JA, Hoarau G, Costa JF, Hogerdijk B, Serrão EA, Billard E, Valero M, Pearson GA, Olsen JL (2011). Evolution and diversification within the intertidal brown macroalgae *Fucus spiralis*/*F. vesiculosus* species complex in the North Atlantic. Molecular Phylogenetics and Evolution 58: 283-296; **(i)** Benhissoune, S., Boudouresque, C.-F., Verlaque, M. (2002). A checklist of the seaweeds of the Mediterranean and Atlantic Coasts of Morocco. II. Phaeophyceae. Botanica Marina 45: 217–230. **(k)** Kazzaz, M., Riadi, H. (1998). Inventaire bibliographique des algues benthiques du littoral marocain I, Chlorophyceae et Phaeophyceae. Acta botánica malacitana, 23: 23-41; **(j)** Russel, G., Hockin, D. C. (1988). The Seaweeds of the lagoon and Khnifiss the Tarfaya coast. In : Dakki M. & Ligny W. for (Eds): The Lagoon and Its Surrounding Khnifiss environment (Province of La'youne, Morocco). Trav. Inst. Sci., Mem. Occasional Papers, p. 37 to 40; **(l)** Bergier, P. (2009). Où voir les oiseaux dans le Sahara Atlantique marocain Go-South Bull. 6, 1-71.

| Location | Coordinates - Lat, Long | Presence/absence | Last recorded (reference to literature or obser. listed in the legend) |
| --- | --- | --- | --- |
| Hondarribia | 43°22'7.27"N, 1°47'32.00"W | Absent | never recorded |
| San Sebastian-Playa de la Concha | 43°19'8.48"N, 1°59'6.01"W | Absent | 1951 (a) |
| San Sebastian- Playa de Ondarreta | 43°19'2.42"N, 2°0'16.59"W | Absent | 1951 (a) |
| Zarautz | 43°17'15.21"N, 2°10'39.11"W | Absent | 1960 (a, b) |
| Getaria | 43°18'2.25"N, 2°12'3.84"W | Absent | 1960 (a, b) |
| Zumaia | 43°17'51.98"N, 2°15'2.30"W | Absent | 1974 (a) |
| Bermeo | 43°25'10.93"N, 2°43'14.26"W | Absent | 1987 (a) |
| Plencia | 43°24'15.94"N, 2°57'2.74"W | Absent | 1988 (a,) |
| El Abra | 43°19'35.47"N, 3° 1'3.28"W | Absent | 1987 (a) |
| Castro-Urdiales | 43°23'0.50"N, 3°13'2.03"W | Absent | 1960 (a, b) |
| **Colindres** | **43°23'36.63"N, 3°27'50.15"W** | **Present** | **2009-2011** |
| Santander- El Puntal | 43°27'58.81"N, 3°45'58.91"W | Present | 2009-2011 |
| Suances | 43°25'30.44"N, 4° 1'56.96"W | Present | 2009-2011 |
| Cóbreces | 43°23'43.35"N, 4°13'22.79"W | Present | 2009-2011 |
| San Vincente della Barquera | 43°23'13.26"N, 4°23'59.47"W | Present | 2009-2011 |
| La Franca | 43°23'37.40"N, 4°34'17.76"W | Present | 2009-2011 |
| Llanes | 43°26'11.13"N, 4°50'9.08"W | Present | 2009-2011 |
| San Martin de Mar | 43°31'21.58"N, 5°23'30.98"W | Present | 2009-2011 |
| Piles | 43°32'39.59"N, 5°38'42.44"W | Present | 2009-2011 |
| Gijón | 43°32'42.21"N, 5°39'39.97"W | Present | 2009-2011 |
| Perán | 43°35'0.36"N, 5°45'20.91"W | Present | 2009-2011 |
| Arnao | 43°34'39.87"N, 5°58'48.47"W | Present | 2009-2011 |
| Concha de Artedo | 43°33'45.32"N, 6°11'2.88"W | Present | 2009-2011 |
| Luarca | 43°32'32.02"N, 6°32'4.33"W | Present | 2009-2011 |
| Puerto de Vega | 43°33'56.86"N, 6°38'43.42"W | Present | 2009-2011 |
| Figueras | 43°32'15.53"N, 7° 1'24.60"W | Present | 2009-2011 |
| Castropol | 43°31'43.95"N, 7° 1'47.28"W | Present | 2009-2011 |
| Foz | 43°33'58.29"N, 7°15'18.59"W | Present | 2009-2011 |
| San Cibrao | 43°41'39.81"N, 7°26'16.51"W | Present | 2009-2011 |
| Viveiro | 43°39'47.21"N, 7°35'52.83"W | Present | 2009-2011 |
| Area Longa | 43°43'49.36"N, 7°40'54.32"W | Present | 2009-2011 |
| Ortigueira | 43°40'59.56"N, 7°51'18.87"W | Present | 2009-2011 |
| Cedeira | 43°39'34.12"N, 8°3'22.87"W | Present | 2009-2011 |
| Ferrol | 43°29'1.61"N, 8°12'43.54"W | Present | 2009-2011 |
| Pasaxe do Pedrido | 43°19'23.45"N, 8°12'27.92"W | Present | 2009-2011 |
| La Coruña | 43°22'10.01"N, 8°24'40.55"W | Present | 2009-2011 |
| Balarés | 43°14'31.96"N, 8°56'42.07"W | Present | 2009-2011 |
| Arou | 43°11'5.83"N, 9°6'25.28"W | Present | 2009-2011 |
| Ria de Lires | 43° 0'3.57"N, 9°15'34.62"W | Present | 2009-2011 |
| Muros | 42°46'25.14"N, 9° 3'10.58"W | Present | 2009-2011 |
| Pesqueira | 42°36'51.53"N, 8°52'57.29"W | Present | 2009-2011 |
| Pontevedra | 42°25'39.87"N, 8°39'9.44"W | Present | 2009-2011 |
| Canido | 42°11'45.17"N, 8°47'48.50"W | Present | 2009-2011 |
| La Guardia | 41°53'55.84"N, 8°52'32.11"W | Present | 2009-2011 |
| Caminha | 41°53'0.23"N, 8°51'14.02"W | Present | 2009-2011 |
| Montedor | 41°44'46.71"N, 8°52'39.37"W | Present | 2009-2011 |
| Viana do Castelo | 41°41'56"N, 8°51'12"W | Present | 2009-2011 |
| Viana do Castelo - Rio Lima | 41°41'47.82"N, 8°51'4.64"W | Present | 2009-2011 |
| Amorosa | 41°38'44.32"N, 8°49'31.14"W | Present | 2009-2011 |
| Apulia | 41°28'53.61"N, 8°46'36.76"W | Present | 2009-2011 |
| Póvoa do Varzim | 41°22'30.22"N, 8°45'53.09"W | Present | 2009-2011 |
| Mindelo | 41°18'37.30"N, 8°44'30.27"W | Present | 2009-2011 |
| Porto | 41° 8'49.85"N, 8°40'3.56"W | Present | 2009-2011 |
| Ria de Aveiro | 40°39'40.24"N, 8°43'44.64"W | Present | 2009-2011 |
| Figueira da Foz | 40° 8'45.69"N, 8°50'28.84"W | Present | 2009-2011 |
| **Alcochete** | **38°45'38.99"N, 8°56'28.43"W** | **Present** | **2009-2011** |
| Vila Nova de Milfontes | 37°43'21.68"N, 8°47'10.81"W | Absent | 2001 (c) |
| Ingrina | 37° 2'46.36"N, 8°52'46.78"W | Absent | 2001-2006 (d) |
| Portimao | 37° 8'30.63"N, 8°31'48.02"W | Absent | 2005 (e) |
| Faro-Ria Formosa | 37° 0'40.81"N, 7°59'28.00"W | Absent | 2008 (f) |
| Tavira | 37° 7'28.26"N, 7°38'35.82"W | Absent | 2001 (g) |
| Ayamonte | 37°12'48.49"N, 7°24'14.03"W | Absent | 2008 (h) |
| El Rompido | 37°13'0.15"N, 7° 7'36.65"W | Absent | 1991 (a) |
| Sanlúcar de Barrameda | 36°47'23.88", 6°21'7.09"W | Absent | 1991 (a) |
| Cádiz-La Caleta | 36°31'58.79"N, 6°18'26.27"W | Absent | 1977 (a) |
| Cádiz-Playa de La Victoria | 36°30'16.63"N, 6°16'42.84"W | Absent | 1977 (a) |
| Cádiz-Puerto Real | 36°31'27.53"N, 6°10'52.61"W | Present | 2009-2011* |
| Tarifa | 36° 0'28.39"N, 5°36'24.72"W | Absent | 1988 (a) |
| Tanger | 35°46'58.87"N, 5°48'13.52"W | Absent | 1962 (i) |
| Azilah | 35°27'58.29"N, 6° 2'23.99"W | Absent | 2002 (i) |
| Lixus | 35°12'8.67"N, 6°6'47.89"W | Absent | 2001 (c) |
| Kenitra | 34°15'58.47"N, 6°35'18.16"W | Absent | 2002 (i) |
| Rabat | 34° 1'45.29"N, 6°49'46.85"W | Absent | 1963 (i, k) |
| Temara | 33°56'50.28"N, 6°56'1.41"W | Absent | 2002 (i) |
| Casablanca | 33°36'33.25"N, 7°39'1.14"W | Absent | 1962 (i) |
| El Jadida | 33°15'10.19"N, 8°30'0.23"W | Absent | 1989 (i, k) |
| Oualidia | 32°44'34.00"N, 9°2'20.53"W | Absent | 1962 (i) |
| Safi | 32°18'51.11"N, 9°15'1.89"W | Absent | 1962 (i) |
| Essaouira | 31°30'36.72"N, 9°46'13.85"W | Absent | 1962 (i) |
| Agadir | 30°25'20.99"N, 9°36'57.25"W | Absent | never reported |
| El Ouatia | 28°28'34.09"N, 11°21'21.08"W | Absent | never reported |
| **Khnifiss Lagoon** | **27°59'40.82"N,12°16'33.98"W** | **Absent** | **1985-1988** (j, l) |
| Boujdour | 26° 7'23.21"N, 14°30'0.18"W | Absent | never reported |
| Daklha | 23°41'58.80"N, 15°55'39.43"W | Absent | never reported |
