# Supplementary figures and images for: Shift happens: trailing edge contraction associated with recent warming trends threatens a distinct genetic lineage in the marine macroalga Fucus vesiculosus
Source: BMC Biol. 2013 Jan 23;11:6. doi: 10.1186/1741-7007-11-6 (PMC3598678; doi:10.1186/1741-7007-11-6)

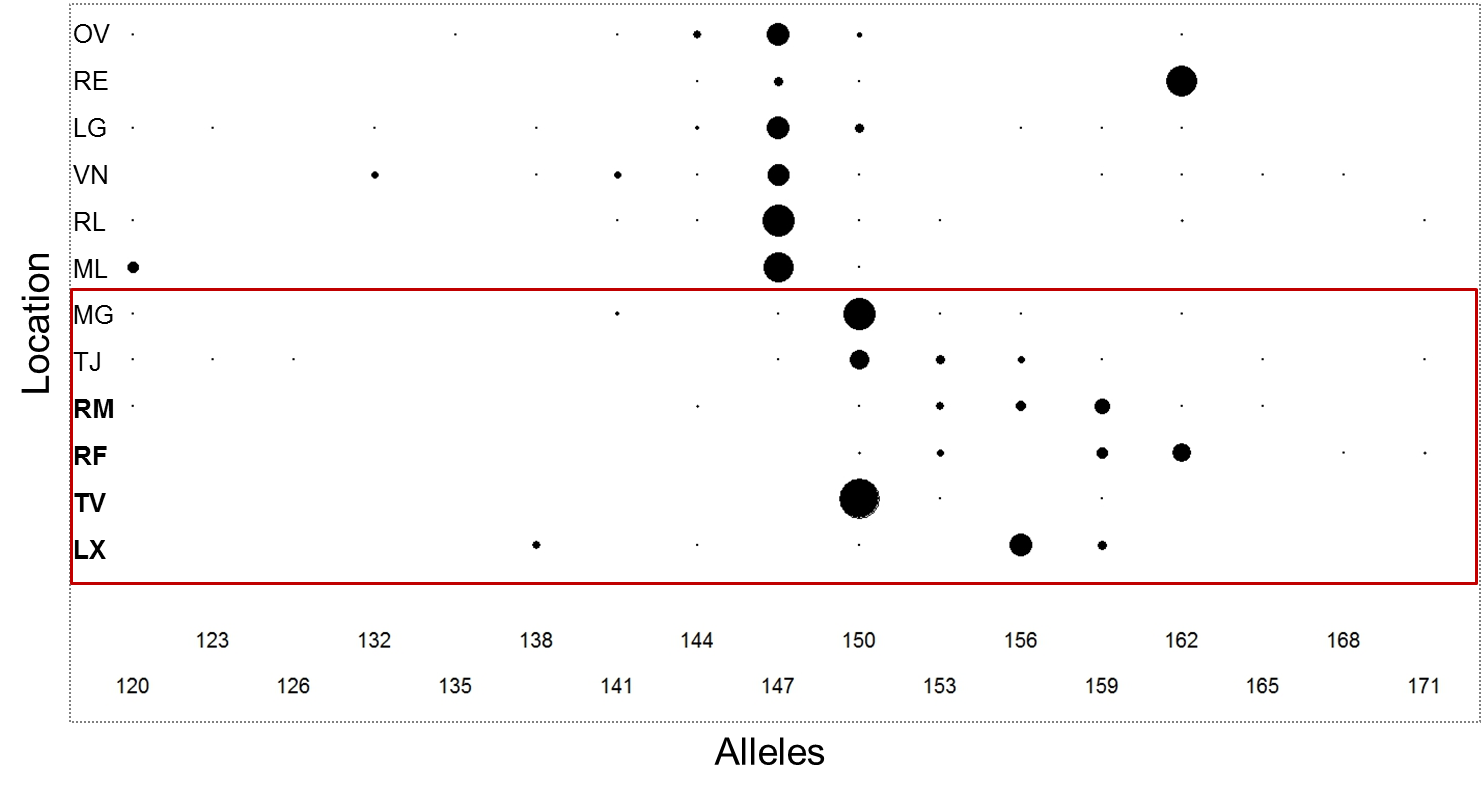

Supplement: Additional file 2 — Allele frequencies for locus L20. Codes correspond to locations in Figure 1, locations belonging to the southern lineage are encircled and extinct populations are shown in bold. The actual values of frequencies are represented by dots of varying diameter: allele codes are indicated on the x axis and population names on the y axis. [file 1741-7007-11-6-S2.TIFF]

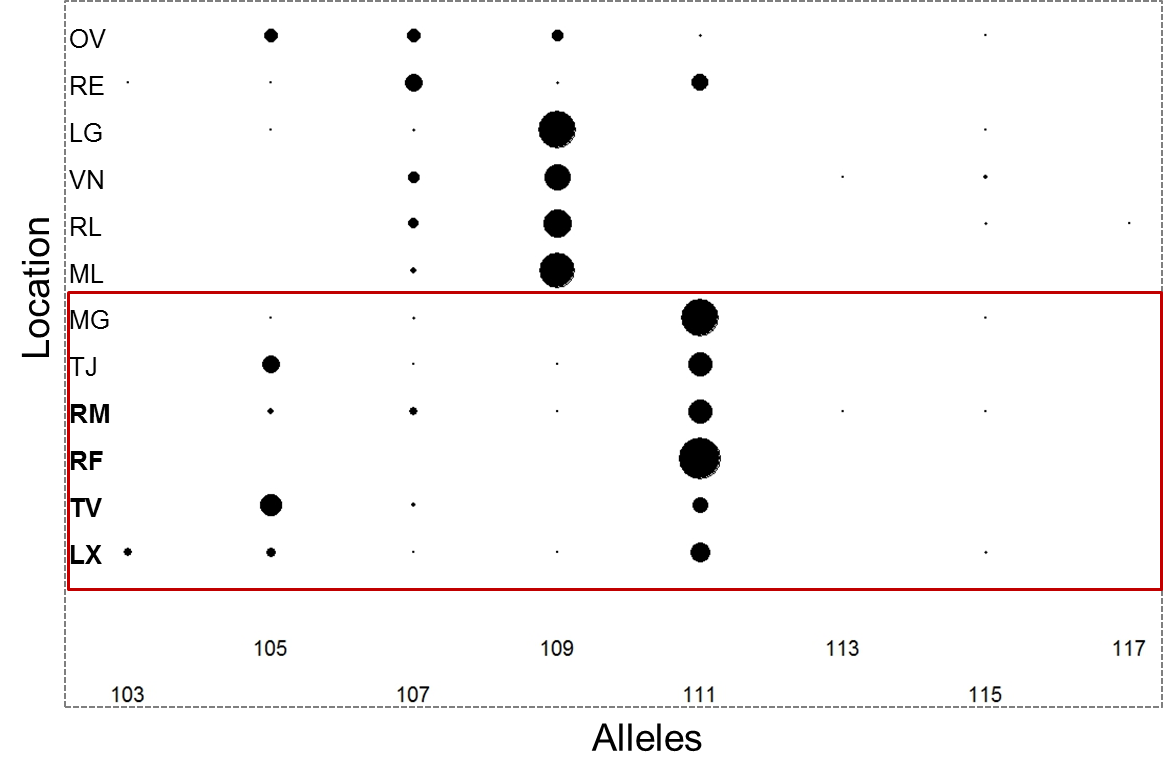

Supplement: Additional file 3 — Allele frequencies for locus L58. Codes correspond to locations in Figure 1, locations belonging to the southern lineage are encircled and extinct populations are shown in bold. The actual values of frequencies are represented by dots of varying diameter: allele codes are indicated on the x axis and population names on the y axis. [file 1741-7007-11-6-S3.TIFF]

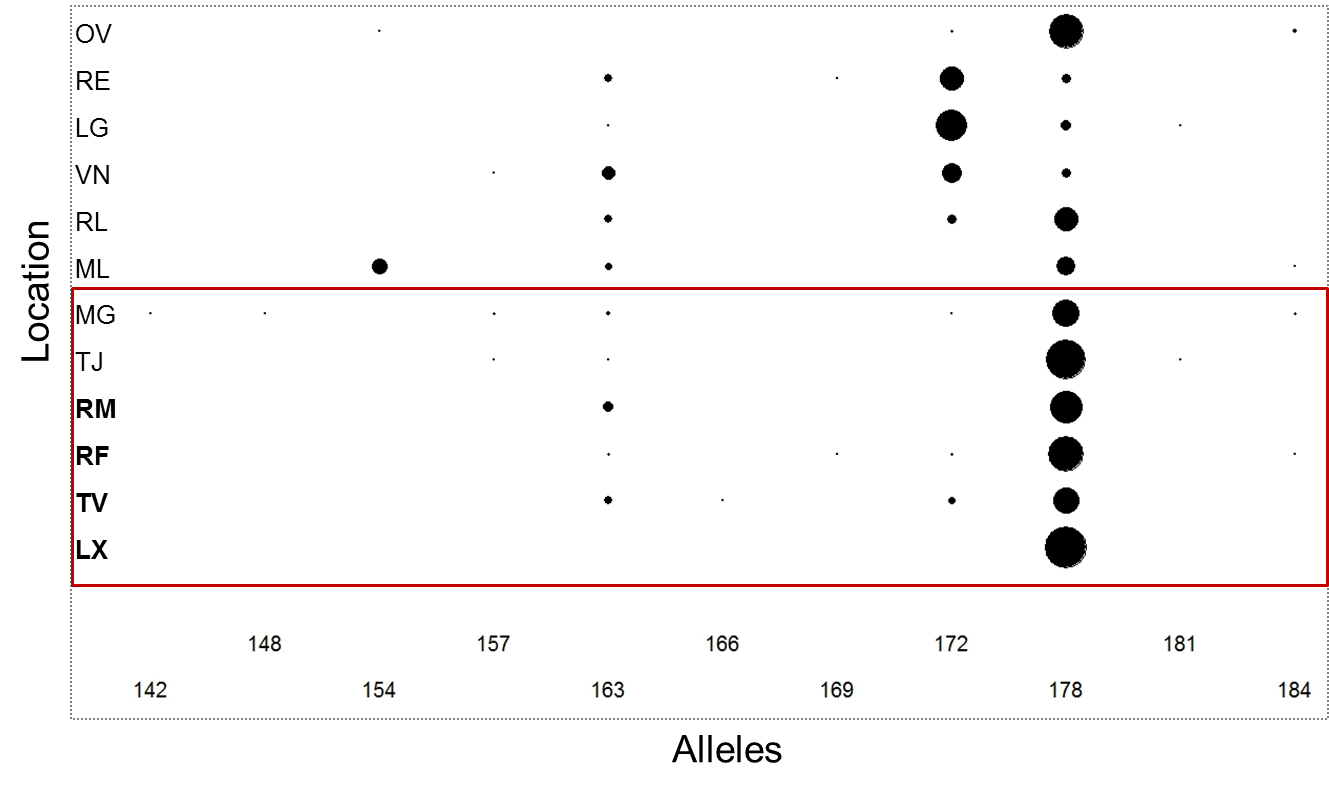

Supplement: Additional file 4 — Allele frequencies for locus L94. Codes correspond to locations in Figure 1, locations belonging to the southern lineage are encircled and extinct populations are shown in bold. The actual values of frequencies are represented by dots of varying diameter: allele codes are indicated on the x axis and population names on the y axis. [file 1741-7007-11-6-S4.TIFF]

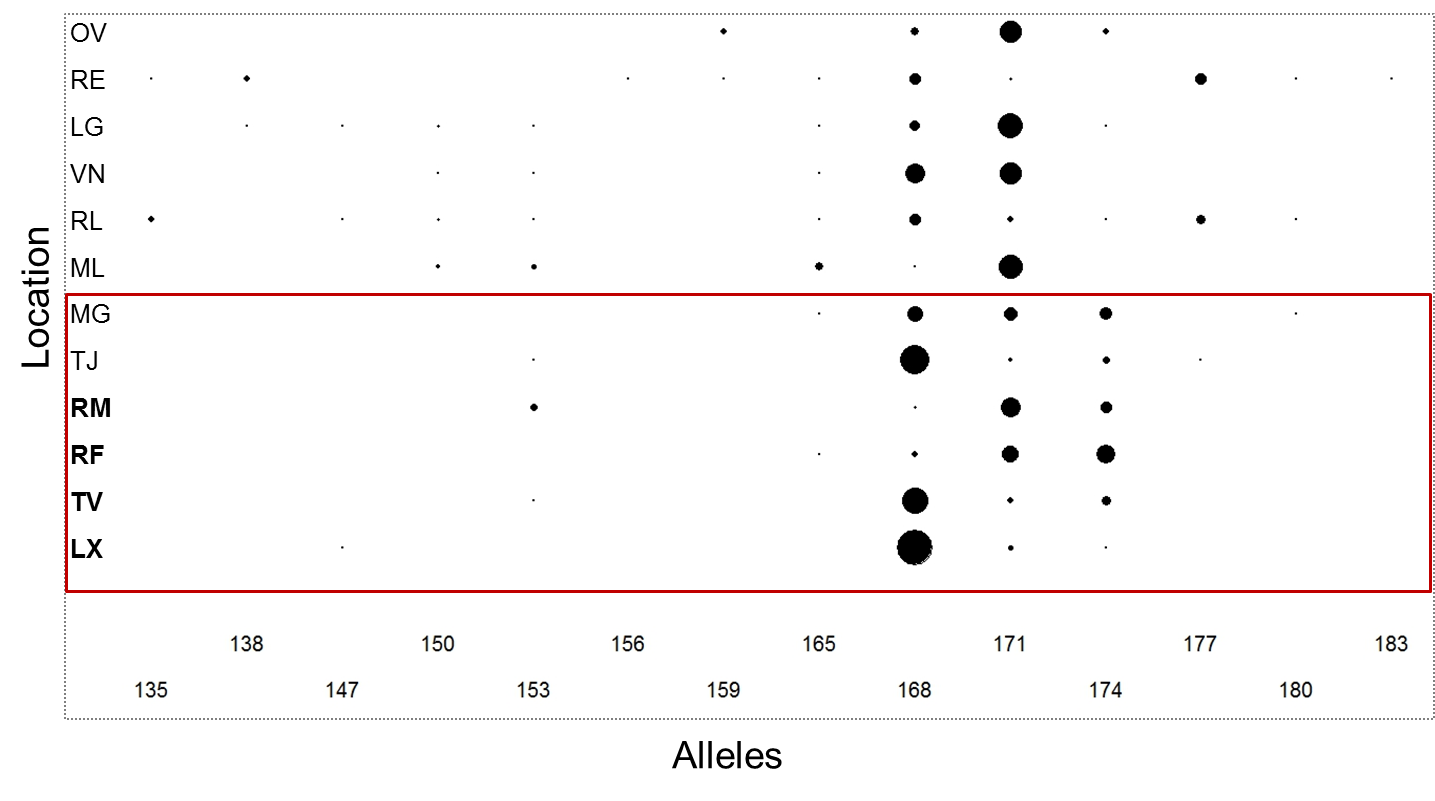

Supplement: Additional file 5 — Allele frequencies for locus L78. Codes correspond to locations in Figure 1, locations belonging to the southern lineage are encircled and extinct populations are shown in bold. The actual values of frequencies are represented by dots of varying diameter: allele codes are indicated on the x axis and population names on the y axis. [file 1741-7007-11-6-S5.TIFF]

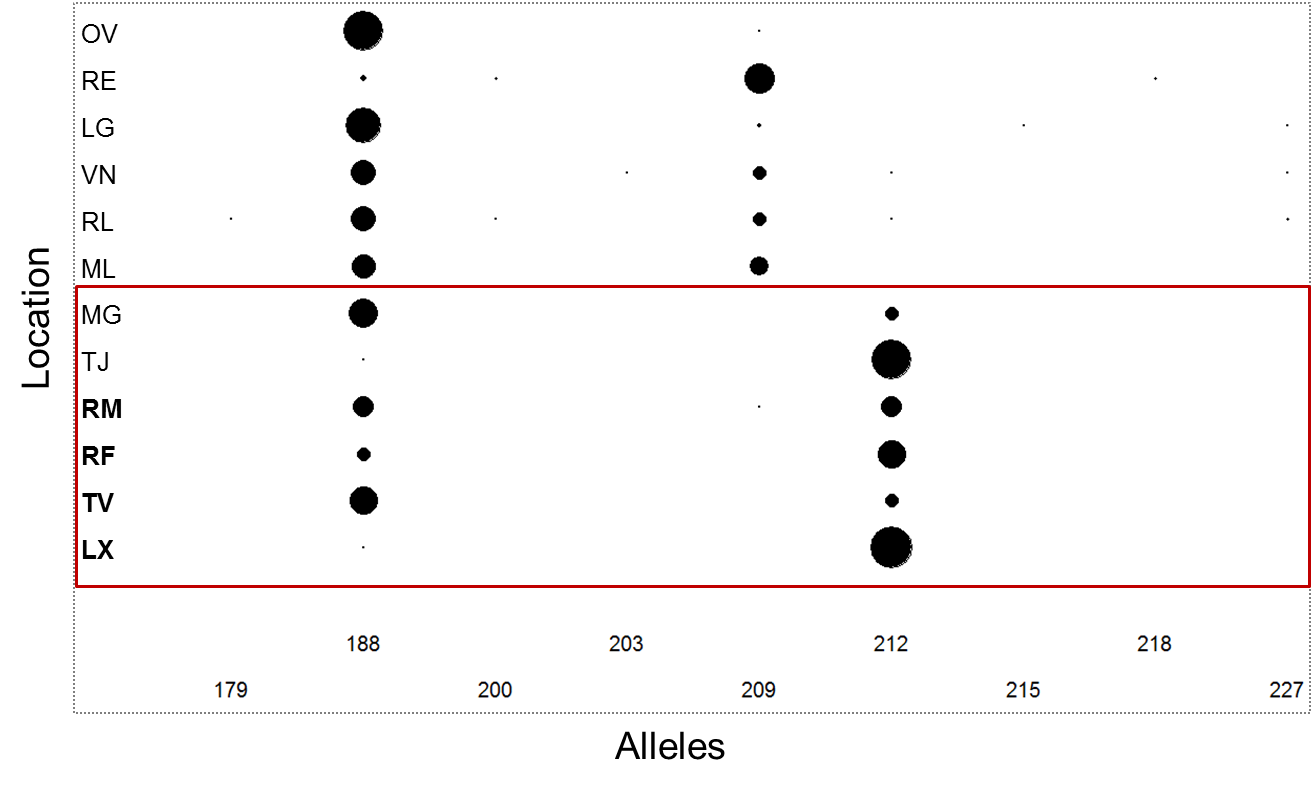

Supplement: Additional file 6 — Allele frequencies for locus L38. Codes correspond to locations in Figure 1, locations belonging to the southern lineage are encircled and extinct populations are shown in bold. The actual values of frequencies are represented by dots of varying diameter: allele codes are indicated on the x axis and population names on the y axis. [file 1741-7007-11-6-S6.TIFF]
