# Supplementary material for: Shift happens: trailing edge contraction associated with recent warming trends threatens a distinct genetic lineage in the marine macroalga Fucus vesiculosus
Source: BMC Biol. 2013 Jan 23;11:6. doi: 10.1186/1741-7007-11-6 (PMC3598678; doi:10.1186/1741-7007-11-6)
Supplement: Additional file 7 — Genetic differentiation between pairs of populations. Codes correspond to locations in Figure 1 and are ordered from north to south, bold characters are extinct populations. Genetic differentiations (FST) were estimated with the estimator θ, and are reported above the diagonal while Jost's DEST are reported below the diagonal. All values are significant at P <0.001 after multiple test correction. [file 1741-7007-11-6-S7.DOCX]

**Table A2 - Genetic differentiation between pairs of populations**

| Location | OV | RE | LG | VN | RL | ML | MG | TJ | **RM** | **RF** | **TV** | **LX** |
| --- | --- | --- | --- | --- | --- | --- | --- | --- | --- | --- | --- | --- |
| OV |  | 0.21 | 0.19 | 0.13 | 0.11 | 0.18 | 0.26 | 0.32 | 0.16 | 0.31 | 0.26 | 0.4 |
| RE | 0.62 |  | 0.28 | 0.16 | 0.2 | 0.3 | 0.33 | 0.38 | 0.25 | 0.3 | 0.33 | 0.43 |
| LG | 0.24 | 0.58 |  | 0.07 | 0.14 | 0.17 | 0.37 | 0.47 | 0.33 | 0.44 | 0.37 | 0.52 |
| VN | 0.25 | 0.44 | 0.1 |  | 0.06 | 0.12 | 0.3 | 0.37 | 0.24 | 0.35 | 0.29 | 0.4 |
| RL | 0.18 | 0.45 | 0.18 | 0.12 |  | 0.1 | 0.33 | 0.39 | 0.26 | 0.37 | 0.32 | 0.43 |
| ML | 0.24 | 0.62 | 0.21 | 0.19 | 0.14 |  | 0.4 | 0.48 | 0.3 | 0.44 | 0.42 | 0.52 |
| MG | 0.35 | 0.65 | 0.54 | 0.55 | 0.56 | 0.63 |  | 0.22 | 0.16 | 0.19 | 0.12 | 0.36 |
| TJ | 0.56 | 0.73 | 0.78 | 0.71 | 0.67 | 0.80 | 0.25 |  | 0.2 | 0.23 | 0.2 | 0.13 |
| **RM** | 0.31 | 0.66 | 0.6 | 0.55 | 0.54 | 0.57 | 0.22 | 0.26 |  | 0.1 | 0.23 | 0.25 |
| **RF** | 0.48 | 0.56 | 0.71 | 0.69 | 0.66 | 0.71 | 0.2 | 0.23 | 0.12 |  | 0.33 | 0.32 |
| **TV** | 0.35 | 0.67 | 0.53 | 0.52 | 0.56 | 0.69 | 0.12 | 0.22 | 0.35 | 0.42 |  | 0.38 |
| **LX** | 0.6 | 0.75 | 0.82 | 0.71 | 0.68 | 0.82 | 0.42 | 0.09 | 0.29 | 0.3 | 0.42 |  |
