# Supplementary material for: Shift happens: trailing edge contraction associated with recent warming trends threatens a distinct genetic lineage in the marine macroalga Fucus vesiculosus
Source: BMC Biol. 2013 Jan 23;11:6. doi: 10.1186/1741-7007-11-6 (PMC3598678; doi:10.1186/1741-7007-11-6)
Supplement: Additional file 8 — Confidence intervals of the genetic differentiation between pairs of populations. Codes correspond to locations in Figure 1 and are ordered from north to south, bold characters are extinct populations. Confidence intervals of genetic differentiation (FST) are reported above the diagonal. [file 1741-7007-11-6-S8.DOCX]

**Table A3 – Confidence intervals of the genetic differentiation between pairs of populations**

| Location | OV | RE | LG | VN | RL | ML | MG | TJ | **RM** | **RF** | **TV** | **LX** |
| --- | --- | --- | --- | --- | --- | --- | --- | --- | --- | --- | --- | --- |
| OV |  | 0.15-0.27 | 0.14-0.24 | 0.1-0.17 | 0.07-0.15 | 0.13-0.22 | 0.22-0.31 | 0.27-0.36 | 0.12-0.2 | 0.26-0.35 | 0.2-0.31 | 0.35-0.45 |
| RE |  |  | 0.22-0.33 | 0.12-0.21 | 0.14-0.25 | 0.24-0.35 | 0.28-0.37 | 0.34-0.42 | 0.20-0.3 | 0.26-0.35 | 0.28-0.37 | 0.38-0.47 |
| LG |  |  |  | 0.05-0.09 | 0.09-0.18 | 0.14-0.21 | 0.33-0.41 | 0.43-0.5 | 0.28-0.37 | 0.4-0.48 | 0.32-0.42 | 0.48-0.57 |
| VN |  |  |  |  | 0.03-0.08 | 0.09-0.14 | 0.28-0.33 | 0.35-0.4 | 0.21-0.28 | 0.32-0.38 | 0.26-0.31 | 0.37-0.43 |
| RL |  |  |  |  |  | 0.07-0.13 | 0.3-0.36 | 0.36-0.42 | 0.21-0.3 | 0.34-0.41 | 0.29-0.36 | 0.4-0.47 |
| ML |  |  |  |  |  |  | 0.36-0.43 | 0.45-0.51 | 0.26-0.35 | 0.4-0.47 | 0.38-0.46 | 0.49-0.56 |
| MG |  |  |  |  |  |  |  | 0.18-0.25 | 0.13-0.19 | 0.15-0.23 | 0.08-0.15 | 0.33-0.4 |
| TJ |  |  |  |  |  |  |  |  | 0.16-0.24 | 0.19-0.27 | 0.15-0.24 | 0.1-0.15 |
| **RM** |  |  |  |  |  |  |  |  |  | 0.06-0.14 | 0.19-0.26 | 0.2-0.29 |
| **RF** |  |  |  |  |  |  |  |  |  |  | 0.29-0.38 | 0.28-0.37 |
| **TV** |  |  |  |  |  |  |  |  |  |  |  | 0.33-0.42 |
| **LX** |  |  |  |  |  |  |  |  |  |  |  |  |
